# Supplementary material for: Growth Hormone (GH) Crosses the Blood–Brain Barrier (BBB) and Induces Neuroprotective Effects in the Embryonic Chicken Cerebellum after a Hypoxic Injury
Source: Int J Mol Sci. 2022 Sep 30;23(19):11546. doi: 10.3390/ijms231911546 (PMC9570246; doi:10.3390/ijms231911546)
Supplement: Supplementary file 1 [file ijms-23-11546-s001.zip › ijms-1911009-supplementary.pdf]

International Journal of
Molecular Sciences

MDPI

Supplementary Material

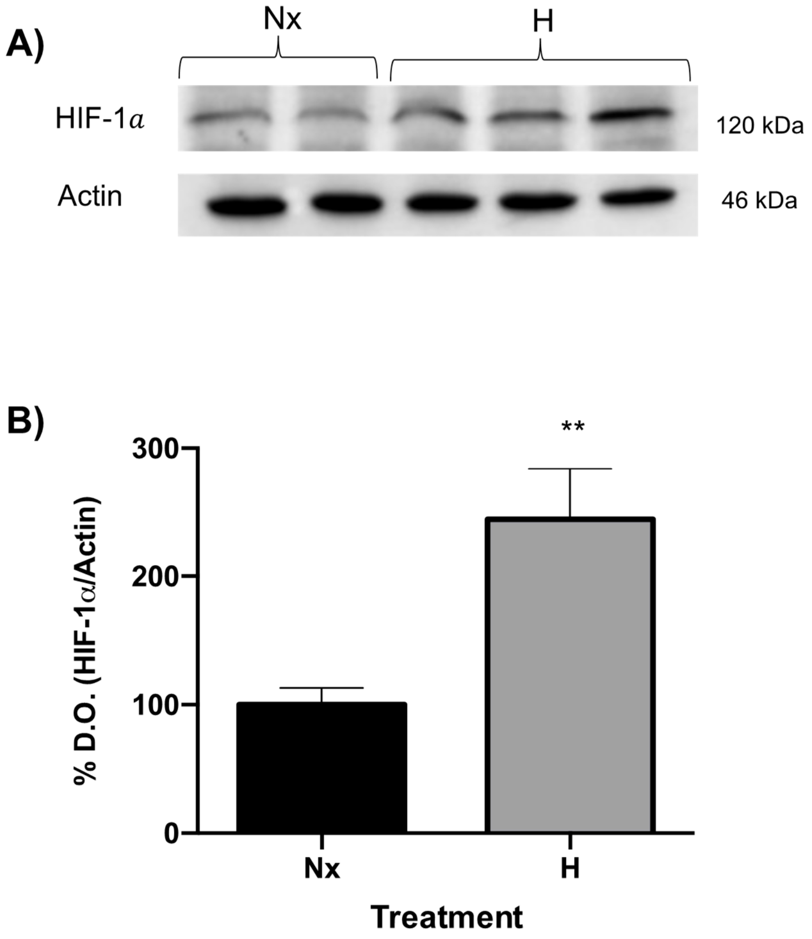

Figure S1. HIF-1α increases under hypoxic conditions in the cerebellum. (A) Representative Western blots showing HIF-1α immunoreactivity in the cerebellum of animals under normoxia (Nx) and hypoxia conditions (H). (B) Densitometric analysis of immunoblots showing the relative proportion of HIF-1α expression in relation to actin. Each bar represent means ± SEM (n = 5). An unpaired Student's t-test was used to compare Nx vs H groups, and asterisks (**) indicate a significant difference (p <0.01).

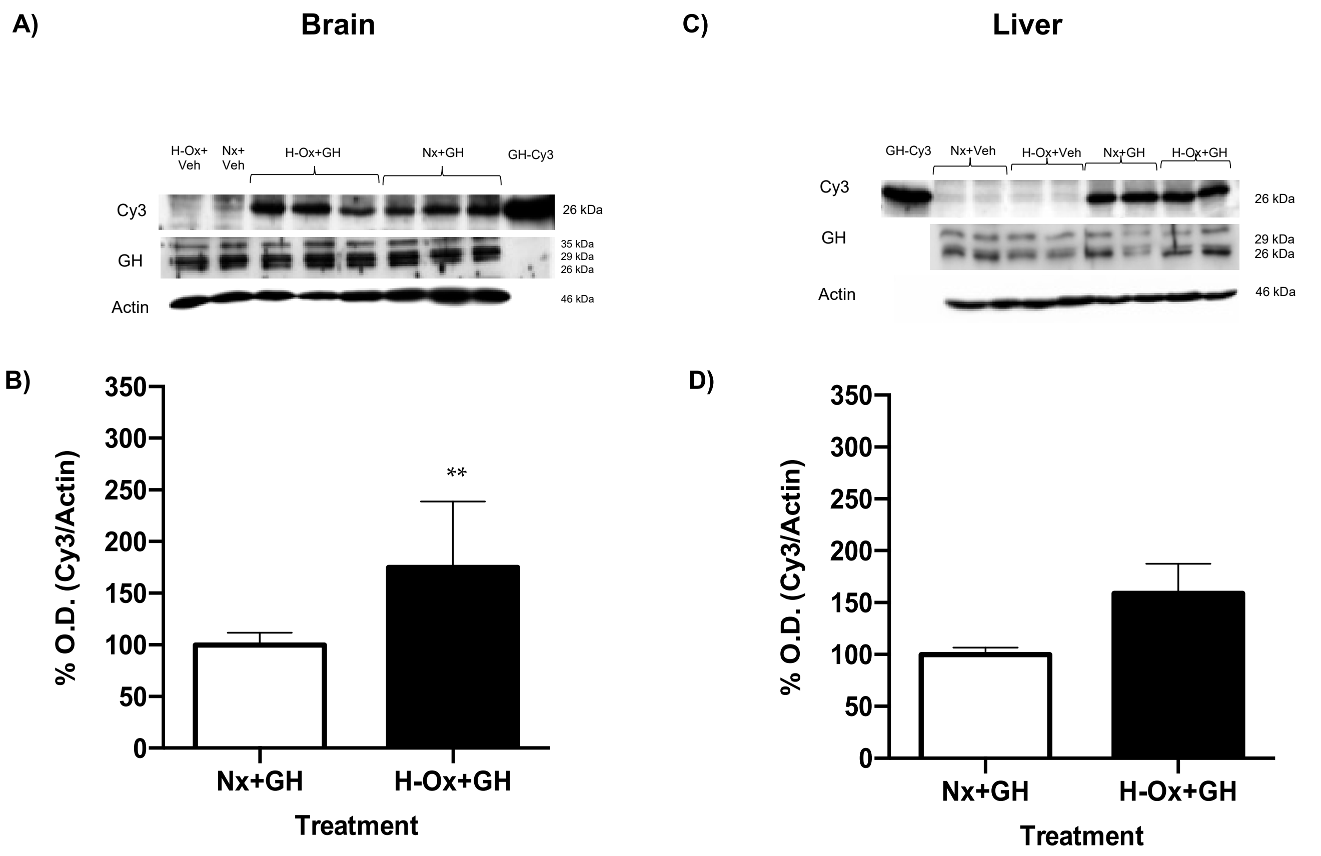

Figure S2. Western blot was used to analyze the integrity of recombinant chicken Cy3-GH after i.v. administration in chicken embryos. Densitometric and immunoblot analysis against Cy3 in (A-B) Brain and (C-D) liver homogenates. Figures A and C correspond to the relative proportion of Cy3-GH under conditions of normoxia and hypoxia. Figure B and D show the band obtained with the antibody against Cy3, which is close to the control (GH) with an approximate molecular weight of 26 KDa and which is absent in animals treated with vehicle. This band was also observed with the antibody against GH. Nx+Veh (n = 3), H-Ox+Veh (n = 3), Nx+GH (n = 6) and H-Ox+GH (n = 6). Each bar represent means ± SEM. An unpaired Student's t-test was used, asterisks (**) indicate a significant difference (p <0.01).

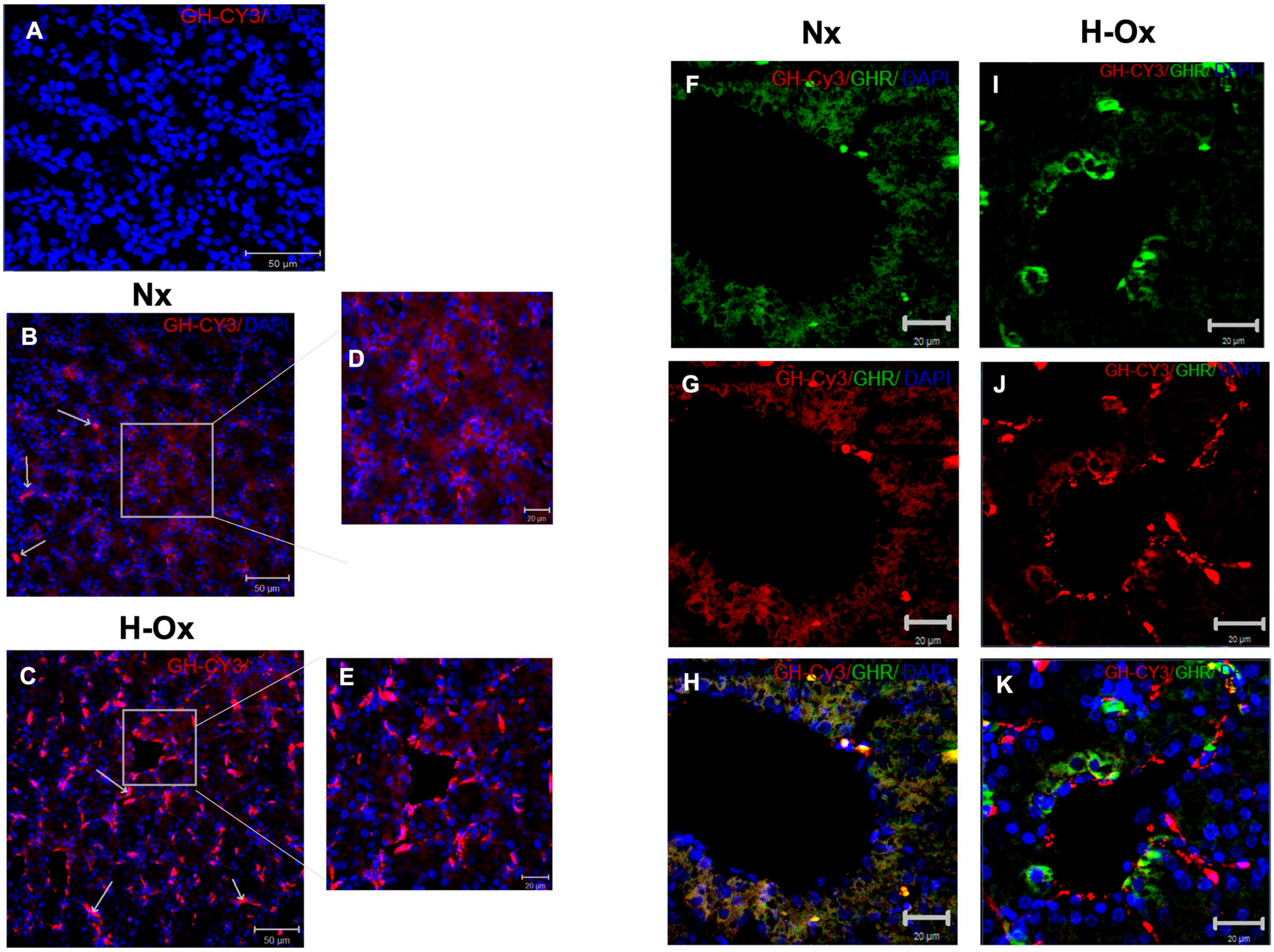

Figure S3. Cy3-GH distribution in the liver. Representative images of Cy3-GH in the liver, images correspond to animals that were administered saline solution under normoxia condition (3A); animals subjected to normoxia with GH-Cy3 (3B and 3D); and animals under hypoxia 24h with Cy3-GH (3C and 3E). The arrows indicate the cells where the labeled hormone was located. GH receptor distribution in the liver. Representative images of immunohistochemistry for GHR in the liver. The images correspond to animals administered with Cy3-GH under normoxia conditions (3F-3H) and hypoxia (3I-3K) separated in the different channels: On the top panel for the GHR, in the middle panel for Cy3-GH, and on the bottom panel for GHR + Cy3-GH + DAPI. The yellow regions are areas where the hormone co-localizes with its receptor.

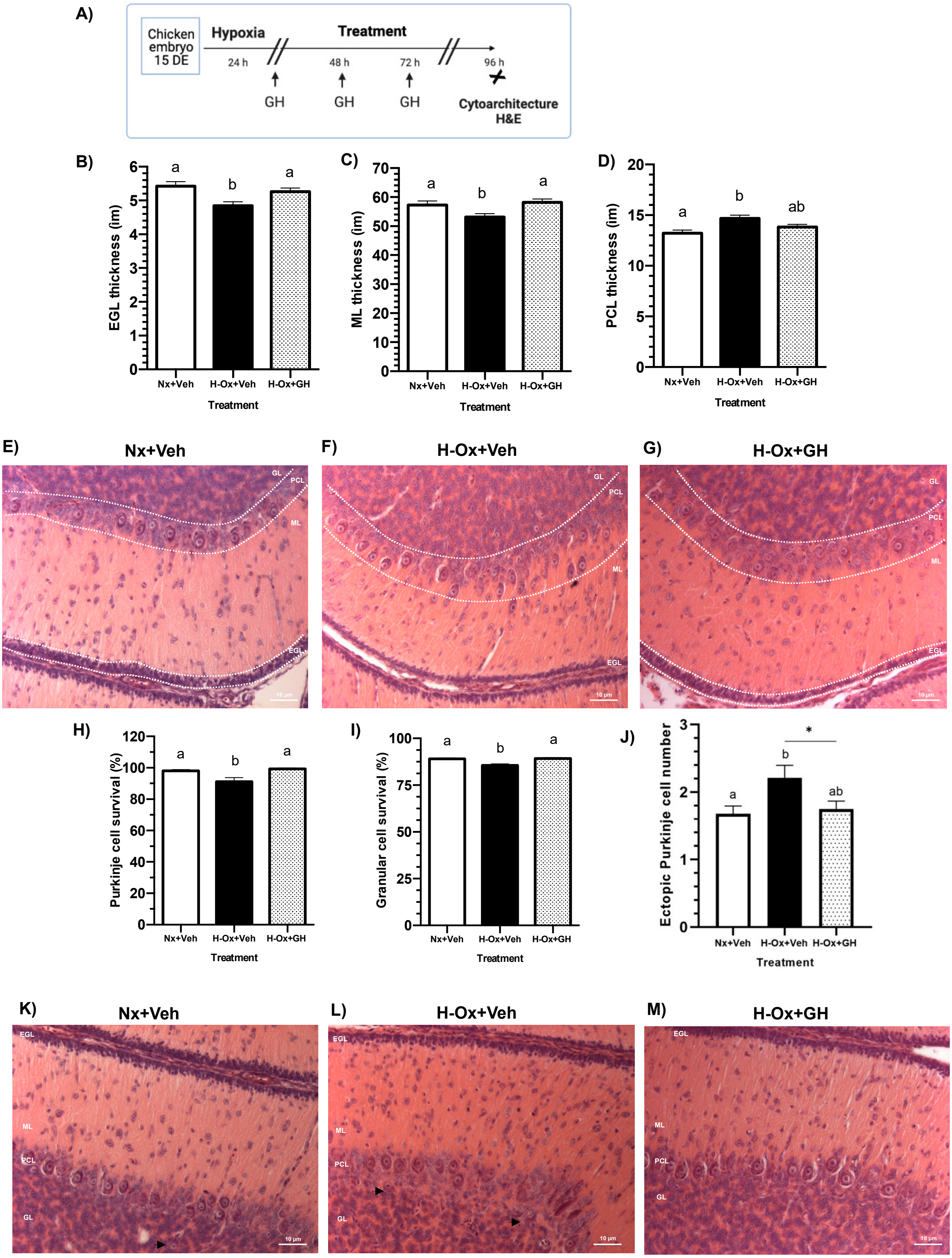

Figure S4. Morphometric analysis showing the regenerative effect of growth hormone after hypoxia injury in chicken cerebellum. (A) Treatment and time-line schematic representation of intravenous injection protocol in the experimental groups. Embryos were treated with three daily doses of recombinant chicken GH (0.15 μg/g). Histological analysis in hematoxylin-eosin stained chicken cerebellar slices obtained from control (Nx+Veh; E,

K), exposed to hypoxia damage (H-Ox+Veh; F, L), or treated with GH (H-Ox+GH; G, M), animals. The thickness of cerebellum was quantified after each treatment: B) external granular layer (EGL), C) Molecular layer (ML) and D) Purkinje cell layer (PCL). The number of Purkinje and granular neurons was quantified in H) Purkinje cell survival (%); I) granular neurons survival % in the ML; and J) Ectopic Purkinje cells. Bars indicate mean ± SEM ($n = 3$ animals per group, 10 fields were quantified per cerebellum/animal). Asterisks indicate significant difference in comparison to control (*, $p < 0.05$) and different letters show differences between experimental groups ($p < 0.05$) as determined by one-way ANOVA for multiple comparisons and Tukey as post hoc test. Scale bar 10 μm.
